# Transcatheter Arterial Chemoembolization Based on Hepatic Hemodynamics for Hepatocellular Carcinoma

**DOI:** 10.1155/2013/479805

**Published:** 2013-03-27

**Authors:** Satoru Murata, Takahiko Mine, Tatsuo Ueda, Ken Nakazawa, Shiro Onozawa, Daisuke Yasui, Shin-ichiro Kumita

**Affiliations:** Department of Radiology, Center for Advanced Medical Technology, Nippon Medical School, 1-1-5 Sendagi, Bunkyo-ku, Tokyo 113-8602, Japan

## Abstract

Hepatocellular carcinoma (HCC) is the sixth most common cancer and the third leading cause of cancer-related deaths in the world. The Barcelona Clinic Liver Cancer (BCLC) classification has recently emerged as the standard classification system for clinical management of patients with HCC. According to the BCLC staging system, curative therapies (resection, transplantation, and percutaneous ablation) can improve survival in HCC patients diagnosed at an early stage and offer potential long-term curative effects. Patients with intermediate-stage HCC benefit from transcatheter arterial chemoembolization (TACE), and those diagnosed at an advanced stage receive sorafenib, a multikinase inhibitor, or conservative therapy. Most patients receive palliative or conservative therapy only, and approximately 50% of patients with HCC are candidates for systemic therapy. TACE is often recommended for advanced-stage HCC patients all over the world because these patients desire therapy that is more effective than systemic chemotherapy or conservative treatment. This paper aims to summarize both the published data and important ongoing studies for TACE and to discuss technical improvements in TACE for advanced-stage HCC.

## 1. Introduction

Hepatocellular carcinoma (HCC) is a major health problem. It is the sixth most common cancer and the third leading cause of cancer-related deaths in the world [1]. In developed countries, 30–40% of patients with HCC are diagnosed at an early stage, when the disease is amenable to treatment approaches such as surgical resection, liver transplantation, and local ablation [2]. However, most patients receive palliative or conservative therapy only. To date, no systemic therapy has improved survival in patients with advanced HCC [3, 4]. A recent study found that administration of sorafenib, a molecular target-based drug, may be an effective treatment that could cause a modest improvement in prognosis [5]. 

In recent years, the Barcelona Clinic Liver Cancer (BCLC) classification has emerged as the standard classification system for clinical management of patients with HCC (Table 1) [6]. According to the BCLC staging system, transcatheter arterial chemoembolization (TACE) is the current standard of care for patients with intermediate-stage disease. Some randomized control studies reported that TACE prolonged survival and allowed control of symptoms in HCC [7–9]. TACE is often recommended for advanced HCC because these patients require therapy that is more effective than systemic chemotherapy or conservative treatment. The aims of this paper are to summarize both the published data and important ongoing studies on TACE, to highlight several problems associated with TACE, and to discuss technical improvements in TACE for HCC. 

## 2. History of Transcatheter Arterial Embolization

Liver circulation is unique because of the dual blood supply by the portal vein and hepatic artery. The portal vein is responsible for 80% of the blood supply to healthy liver tissue. In contrast, 99% of the blood supply to hepatic tumors is delivered by the hepatic artery. Based on this observation, transcatheter arterial embolization (TAE) for HCC is appropriate for patients for whom surgical or percutaneous ablative treatment is contraindicated. 

TAE, first described by Doyon et al. in 1974 [10], is a treatment method in which embolic agents are injected into the hepatic artery to induce ischemic necrosis of a tumor. In the 1980s, iodized oil (Lipiodol Ultrafluide, Laboratoire Guerber, Aulnay-Sous-Bois, France) injected into the hepatic artery was found to selectively accumulate and be retained for long periods in hypervascular hepatic tumors. However, TACE, a recently introduced interventional radiological treatment for HCC, involves injection of anticancer drugs and iodized oil into the hepatic artery, followed by the administration of embolic agents [11, 12]. The antitumor effect of TACE is greater than that of either anticancer drugs [13] or iodized oil [14, 15] administered alone. Moreover, TACE can be performed in many patients who are not suitable candidates for surgical or percutaneous ablation. 

## 3. The Comparative Efficacy of Anticancer Agent-Iodized Oil Suspensions and Emulsions in TACE

Iodized oil is used as an embolic agent and a carrier of anticancer drugs in TACE. Mixtures of anticancer drugs and iodized oil are classified as emulsions (oil with saline and drugs) or suspensions (drugs in oil) [16, 17]. Comparative studies of suspensions versus emulsions in TACE, with cisplatin powder [18] or epirubicin [19] serving as anticancer agents for treatment of rabbit VX2 liver tumors, demonstrated that a suspension is superior to an emulsion for drug delivery and antitumor effect. Several factors are thought to explain these findings. In an emulsion, most of the powdered drug is contained in the unstable aqueous phase [12, 20]. The drug undergoes rapid dilution into the blood, elimination from the hepatic tissue, and excretion by the kidneys. Conversely, in a suspension, the powder is directly mixed with the oily phase and is distributed in similar fashion to iodized oil alone in the portal venules and sinusoids over a 24 h period [21, 22]. As a result, suspensions show a longer anticancer drug release time at the tumor border and higher continuous drug concentrations. The longer tissue drug activity period associated with the use of suspensions produces superior antitumor effects, as evaluated by the growth ratio and the results of histopathological investigations [16, 17, 23, 24]. Some problems exist, however. Since most anticancer drugs are hydrophilic, stable suspensions in oil are not possible. The viscosity of a suspension is higher than that of an emulsion; that is, tumor accumulation of lipiodol with a suspension is less than that with an emulsion. This necessitates the use of a device or an agent that can allow a suspension to be used as an antitumor material.

## 4. How to Improve the Efficacy of TACE?

Histopathological investigations of HCCs resected after TACE have shown that the most viable tissue is located at the periphery of the tumor [25]. The efficacy of TACE is limited by the dual (i.e., arterial and portal) blood supply of liver tumors, which makes it impossible to deliver anticancer agents to the entire tumor area or to achieve sufficient tumor ischemia without irreversible damage to the surrounding normal liver parenchyma. Some researchers [26, 27] have reported that superselective TACE is useful for treatment of small HCCs in conjunction with percutaneous ablation because it can embolize both the tumor and an area of the surrounding normal parenchyma. For large liver tumors, however, the therapeutic options are limited to techniques that result in complete necrosis of the tumor.

### 4.1. Hemodynamics of Dual Hepatic Blood Supply

To obtain complete necrosis of the tumor including the periphery, several researchers [28–30] have investigated the hemodynamic changes occurring in the liver and tumors during hepatic vein balloon occlusion by using computed tomography during hepatic arteriography (CTHA) and arterial portography. These reports demonstrated that the occluded area is supplied with arterial blood alone [29, 30] and suggested that adequate embolization may be obtained during TACE with arterial control alone. Since balloon occlusion of the segmental hepatic vein eliminates the possibility of dual blood supply and allows only arterial supply, TACE performed using this technique can sufficiently embolize both the tumor and the surrounding liver parenchyma. We have performed TACE under balloon occlusion of the hepatic vein for more than 70 patients with advanced HCC, but because of the complex veno-venous communications in the liver, only 30% of the patients could benefit from the procedure [29]. Therefore, sufficient embolization of large liver tumors using the hepatic vein balloon occlusion technique is difficult because these tumors have complex venous drainage, or because the hepatic veins assume the role of draining veins despite single hepatic vein occlusion [29, 31].

When portal venous blood flow is decreased gradually or stopped due to tumor thrombus, thromboembolus, or compression of the portal vein, the affected parenchyma appears as a hyperattenuated area with straight borders on CTHA [32–36]. This appearance is similar to that seen with hepatic vein occlusion. These findings suggest that hepatic arterial blood flow is increased mainly through the peribiliary plexus [36–38]. Only one study [39] investigated hepatic hemodynamic changes under acute balloon occlusion of the portal vein by using single-level dynamic CTHA and reported several phenomena. First, a demarcated hyperattenuated area of the liver parenchyma was noted in the distribution of the occluded portal vein branch, and the attenuation of this area was significantly higher than that of the nonoccluded area (*P* < 0.01). Second, the balloon-occluded portal branch enhancement appeared to result from arterioportal communications in 15 of 16 patients (94%). These findings suggest that when portal venous flow stoppage occurs chronically or acutely, hepatic arterial blood flow is increased. This phenomenon is known as the hepatic artery buffer response; the variations of blood flow observed are due to the degree of clearance of an intrahepatic arterial vasodilator (adenosine) to which the hepatic artery is very sensitive. Indeed, adenosine has been shown to be a potent vasodilator of the hepatic artery [40–42]. Finally, there is little anatomical variation in portal veins or porto-portal venous anastomosis. These facts indicate that sufficient embolization may be obtained, even in large liver tumors, by TACE under temporary occlusion of a portal vein branch. We started TACE under balloon occlusion of the corresponding portal vein for large HCCs 4 years ago. The antitumor effects of this treatment have been promising, and we plan to report our experience with TACE in the near future. 

### 4.2. TACE with Warmed Suspension

As mentioned above, comparative studies of suspensions and emulsions demonstrated that a suspension is superior to an emulsion for drug delivery and antitumor effect during the treatment of rabbit VX2 liver tumors with TACE [12, 18–20]. However, tumor uptake of suspensions is poor, most likely due to their high viscosity [43], and the clinical outcome of treatment with suspensions is less satisfactory than that of treatment with emulsions [43, 44]. The viscosity of iodized oil has a negative correlation with temperature. Viscosity exceeds 50 mPa·s at 20°C, but it decreases to 22 mPa·s or 12 mPa·s at 40°C or 60°C, respectively (unpublished data). This suggests that tumor uptake of a suspension may be improved by warming it to reduce viscosity and that high temperatures are needed to obtain a good antitumor effect when suspensions are used as antitumor materials. 

We designed a syringe warmer 2 years ago to maintain suspensions at a high temperature and started treating HCC with warmed suspensions in January 2011. We obtained significant antitumor effects without major adverse events (paper currently submitted for publication). A prospective, comparative study of TACE with warmed suspension at 50°C is under way, and the results will be reported in the near future.

## 5. How to Perform TACE for Advanced HCC?

Contraindications to TACE have generally included severe synthetic liver dysfunction as indicated by Child-Pugh score, increased serum bilirubin level, impaired hepatopetal flow due to portal hypertension or extension of portal venous tumor thrombus, and significant arterioportal or arteriohepatic vein shunts. With the increasing prevalence of HCC, an increasing number of patients with one or more contraindications could deny treatment. Although systemic treatment with the multikinase inhibitor sorafenib is available and recommended to patients with advanced-stage HCC, some efficient methods of TACE exist for treating HCC with significant arterioportal or arteriohepatic vein shunts. 

### 5.1. TACE for HCC with Significant Arteriohepatic Vein Shunt

Microscopic arteriovenous shunts are usually present in HCC [45]. HCC tends to spread in the portal veins and, to a lesser extent, in the hepatic vein [45], and involvement of intraportal and hepatic veins allows arteriovenous shunts to develop. These shunts represent the main impediment to successful TACE because anticancer drugs or mixtures of iodized oil and anticancer drugs easily pass through them [46]. Conventional TACE is not effective for HCC patients with hepatic arteriovenous shunts and may even be harmful due to the possibility of pulmonary embolism [47–49]. These patients require an effective low-risk treatment option. 

**Figure 1:**
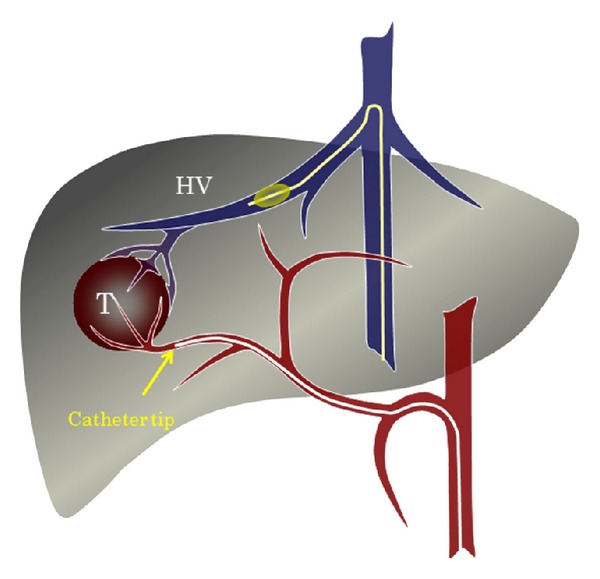
Schema of TACE under hepatic vein occlusion for HCC with significant arteriovenous shunts. T: tumor; HV: hepatic vein.

**Figure 2:**
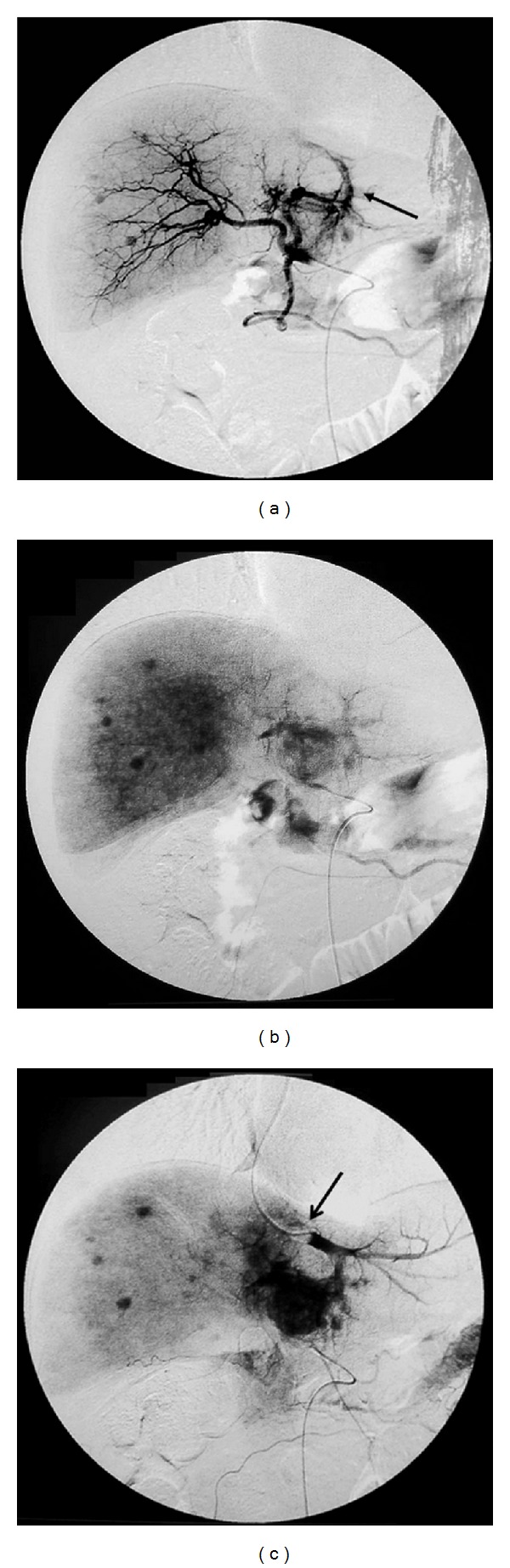
Multiple HCCs with significant arterio-left hepatic vein shunt in a 71-year-old man. Common hepatic arteriography ((a): arterial phase, (b): venous phase) reveals multiple HCCs with significant arterio-left hepatic vein shunts ((a), arrow). Common hepatic arteriography under balloon occlusion of the left hepatic vein demonstrates a dense opacified tumor (c). Arrow of (c) indicates the balloon in the left hepatic vein.

Radiofrequency ablation may be a useful treatment for HCCs smaller than 3 cm in diameter. However, most HCCs with intratumoral arteriohepatic vein shunts are large, and we could not perform radiofrequency ablation in these cases. To overcome this limitation, we attempted TACE of the feeding arteries with balloon occlusion of the corresponding draining hepatic vein, which was monitored by angiography and CT [31] (Figure 1). We performed the procedure using the following protocol. If the target HCC was located in the lateral segment in the liver, we recommended TACE with balloon occlusion of the left hepatic vein (Figure 2). If, however, the target HCC was located in the right or middle lobe of the liver, we recommended TACE with balloon occlusion of 2 hepatic veins, right and middle, in most cases [31]. TACE with balloon occlusion of the corresponding hepatic vein achieves both significant tumor growth control and elimination of the intratumoral shunts [31]. After this modified procedure, conventional TACE can be performed for treatment of residual HCC.

### 5.2. TACE for HCC with Significant Arterioportal Vein Shunt

HCC is frequently associated with arteriovenous shunts, which are mainly arterioportal shunts in nature. Kojiro [50] analyzed 106 resected HCCs less than 2 cm in diameter and found that nodular-type HCC was associated with microscopic portal invasion in up to 25% of cases. Although the presence of small arterioportal shunts does not necessarily preclude TACE therapy for unresectable HCC, larger arterioportal shunts do interfere with TACE because anticancer drugs, either alone or mixed with iodized oil, easily pass through the shunts [47, 48]. Conventional TACE causes extensive embolization of the portal vein and can induce extensive ischemia of nontumorous liver parenchyma in HCC patients with significant arterioportal shunts [46]. The presence of arterioportal shunts can also lead to liver dysfunction and portal hypertension, resulting in potentially life-threatening conditions such as rupture of gastroesophageal varices [51–54], refractory ascites, or hepatic encephalopathy.

**Figure 3:**
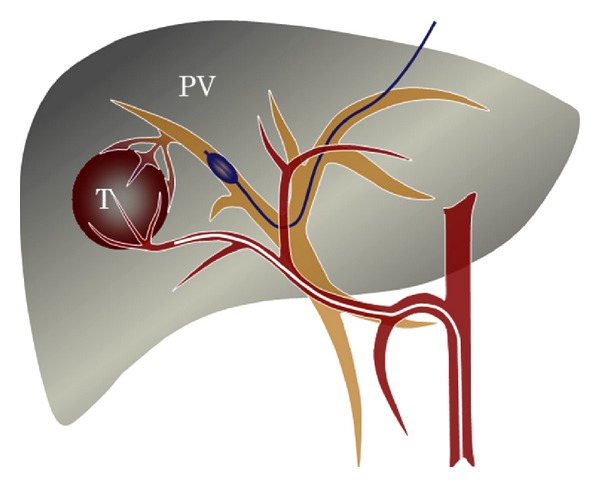
Schema of TACE under portal vein occlusion for HCC with significant arterioportal shunts. T: tumor; PV: portal vein.

Attempts have been made to treat significant arterioportal shunts by embolization of the hepatic arteries with materials such as gelatin sponges or coils, and these approaches have yielded good short-term results [55, 56]. However, these treatments do not eradicate HCC and therefore contribute little to patient survival [55, 56]. A safe and effective therapeutic protocol for HCC with arterioportal shunts remains to be established [12, 57]. We attempted to overcome this complication by performing TACE of tumor-feeding arteries with occlusion of the corresponding portal vein. This method of TACE during portal vein occlusion (TACE-PVO) [58] was designed to permit treatment of HCC with significant arteriohepatic vein shunts as mentioned above [31]. We modified the technique by concurrently performing percutaneous transhepatic portography after evaluation of hemodynamic changes in the liver with portal vein occlusion [39]. This technique is illustrated in Figure 3 and is described as follows. We first selected a puncture point appropriate to avoid tumor penetration using postcontrast CT images. An intrahepatic portal branch was punctured under ultrasonographic guidance by using an 18-gauge percutaneous transhepatic cholangiography needle. For TACE-PVO, a 5- or 8-French balloon catheter was advanced into the portal vein branch identified via direct portography and hepatic arteriography with the arterioportal shunt. The balloon catheter was inflated, and a mixture of lipiodol (up to 15 mL) and anticancer agents, as an emulsion or suspension, was injected via the target feeder artery until reflux into the hepatic artery was confirmed. Particles of gelatin sponge were then immediately injected into the feeder artery until the target hepatic artery was occluded.

The effectiveness of TACE-PVO for arterioportal shunts was ascribed to balloon occlusion of the corresponding portal veins, resulting in adequate embolization of the entire tumor, including the portions involving arterioportal shunts. Consequently, TACE-PVO may prevent the development of collateral anastomoses to arterioportal shunts. 

**Figure 4:**
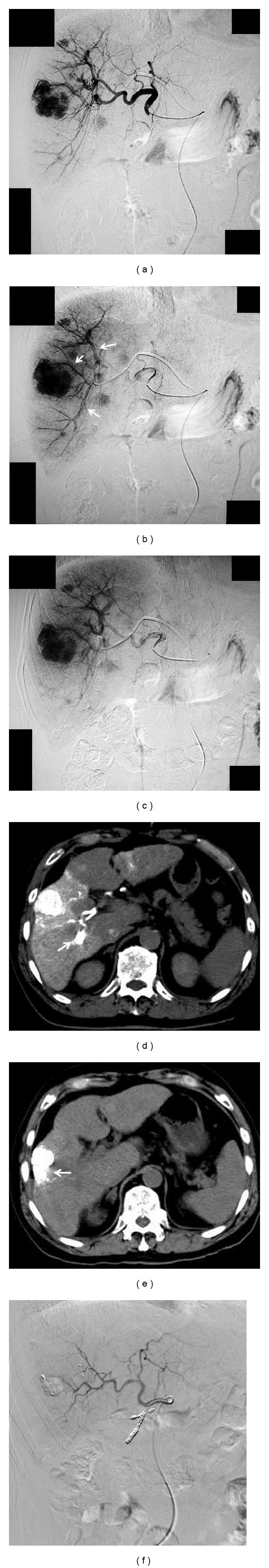
Multiple HCCs with significant arterioportal shunts in a 58-year-old man. Proper hepatic arteriography ((a): early arterial phase, (b): late arterial phase) reveals multiple HCCs with significant arterioportal vein shunts ((b), arrows). (c) indicates proper hepatic arteriography under balloon occlusion of the anterior segmental portal vein. CT during right hepatic arteriography before TACE shows a well-enhanced HCC in the S5. Arrow of (d) indicates a balloon. Precontrast CT one month after TACE-PVO demonstrated a dense lipiodol deposit HCC (e). Lipiodol retains in both HCC and surrounding liver parenchyma ((e), arrow). Common hepatic arteriography 12 months after TACE-PVO reveals that arterioportal shunts and hypervascular tumors are no longer evident (f). The patient first received 2 sessions of conventional TACE for residual HCCs. Reservoir placement was performed 9 months after TACE-PVO. The patient is alive for 4 years after TACE-PVO.

We conducted a comparative study of standard TAE or TACE versus TACE-PVO for HCC with significant arterioportal shunts [58]. This study was a prospective, but not randomized controlled study. The study subjects were fundamentally differentiated only by patients' choice of treatment. We found that TACE-PVO was significantly better (*P* = 0.009) than standard TAE for AP-shunt treatment, and subsequent angiographic findings suggested the superiority of TACE-PVO (*P* = 0.028). Antitumor response (*P* = 0.002) and patient outcome (*P* = 0.032) were significantly better in the TACE-PVO group than in the standard treatment group. Furuse et al. [56] reported that HCC patients with significant arterioportal shunts due to portal vein tumor thrombosis who received embolization had 1- and 2-year survival rates of 12% and 0%, respectively. In our study, 1- and 2-year survival rates in the standard treatment group were 28.6% and 0%, respectively, a result similar to that obtained by Furuse et al. [56]. With TACE-PVO therapy, however, we obtained both a good target tumor response and dramatic improvement in the arterioportal shunts (Figure 4), with favorable 1-, 2-, and 3-year survival rates of 85.7, 64.3, and 42.9%, respectively [58]. Survival appeared to be markedly better than that in the previous series. 

To our knowledge, no effective treatment for both HCC and significant arterioportal shunts has been previously reported. At present, we have performed TACE-PVO on 31 patients with significant arterioportal shunts at our institution, and have obtained good tumor response and prolonged survival. Randomized controlled multicenter trials would be necessary to further explore differences in quality of life and to assess the effects of TACE-PVO on short- and long-term outcomes.

### 5.3. Combination Therapy of TACE and Sorafenib for Advanced HCC

Advanced HCC is usually characterized as a hypervascular tumor. TACE causes both tumor hypoxia and longer active period for any anticancer drug remaining in the tumor tissues. However, TACE also induces a posttreatment surge of angiogenic factors such as vascular endothelial growth factor, which can occur as early as few hours after TACE [59]. This process may contribute to the revascularization of tumors, thus reducing the efficacy of TACE [60, 61]. Combining sorafenib, an antiangiogenic drug, with TACE may potentially improve treatment outcomes [62], and several studies have evaluated the efficacy and safety of combined treatment involving antiangiogenic agents and TACE [63–65]. The dosing schedule of antiangiogenic drugs in relation to TACE is a key factor in the therapy. A randomized phase III study comparing sorafenib with placebo starting at 1–3 months after TACE failed to show a survival benefit [63]. In contrast, a single-arm phase II study with sorafenib starting at 1 week after TACE reported a disease control rate of 95% according to the response evaluation criteria for solid tumors [64]. Abou-Alfa postulated that early post-TACE exposure to antiangiogenic agents may be associated with better clinical outcome [65]. Two years ago, we started a phase II study on the use of sorafenib in combination with TACE in patients with inoperable HCC. In the clinical trial, potential candidates for TACE were started on sorafenib 1 week prior to the procedure. Sorafenib was withheld for 24 h before TACE and, in absence of grade 3 adverse events from the procedure, restarted 24 h after completion. Half of the patients have dropped out of the trial, mainly due to grade 3 adverse events involving liver function or skin. Knowledge of optimal scheduling of antiangiogenic agents with TACE is essential to the improvement of patient prognosis.

## 6. Conclusion

Approximately 50% of patients with HCC are candidates for systemic therapy. Prognosis for this group of patients is extremely poor, and their median overall survival period without treatment is less than 8 months. In this paper, we have introduced the efforts of many researchers to improve the treatment outcomes of patients with intermediate- or advanced-stage HCC. TACE based on hepatic hemodynamics may improve patient outcomes, and TACE-PVO can improve survival for patients with advanced-stage disease. TACE with warmed anticancer drug suspension may also improve target tumor response, and combination treatment with antiangiogenic agents and TACE may be the next generation of TACE-based therapy. In conclusion, the evolution of therapy continues to improve the prognosis of patients with HCC. 

## Figures and Tables

**Table 1:** BCLC classification in patients diagnosed with HCC.

Stage	Description*
Very early	PS 0, Child-Pugh A, single HCC < 2 cm
Early	PS 0, Child-Pugh A-B, single HCC or 3 nodules < 3 cm
Intermediate	PS 0, Child-Pugh A-B, multinodular HCC
Advanced	PS 1-2, Child-Pugh A-B, portal neoplastic invasion, nodal metastases, distant metastases
End-stage	PS > 2, Child-Pugh C

*PS: performance status.

This classification is due to [6].
